# Determinants of unsuccessful tuberculosis treatment outcome in Southwest Ethiopia regional state public hospitals, 2022: a multi-center case control study

**DOI:** 10.3389/fpubh.2024.1406211

**Published:** 2024-10-22

**Authors:** Solomon Berihe Hiluf, Abebe Abera, Mesfin Bahiru, Birhanu Kassie

**Affiliations:** ^1^Department of Nursing, Mizan Aman Health Science College, Mizan Aman, Ethiopia; ^2^Department of Nursing, Institute of Health, Faculty of Health Sciences, School of Nursing, Jimma University, Jimma, Ethiopia; ^3^Department of Biomedical, College of Medicine and Health Science, Mizan Tepi University, Mizan Teferi, Ethiopia

**Keywords:** tuberculosis, unsuccessful treatment outcomes, determinants, Ethiopia, case–control

## Abstract

**Background:**

Tuberculosis is the major cause of morbidity, and it is one of the top ten causes of death globally. In Africa, the overall pooled estimate of unsuccessful tuberculosis treatment outcome was reported to be 21.1%, which is above the World Health Organization defined threshold of 15%. Unsuccessful treatment outcomes result in drug resistance, prolonged periods of infection, and increased morbidity and mortality. Therefore, this study aimed to assess determinants of unsuccessful treatment outcomes among tuberculosis patients in Southwest Ethiopia regional state public hospitals, in 2022.

**Method:**

A retrospective unmatched case–control study was employed by recruiting 570 study units (190 cases and 380 controls) in three randomly selected public hospitals from 1 August 2022 to 21 August 2022. Data were collected by using a data extraction checklist adapted from tuberculosis registration logbook. Bivariate and multivariate logistic regression models were employed. A predictor variable with a *p*-value of less than 0.05 in the multivariate logistic regression model was taken as statistically significant. The odds ratio and 95% confidence level were used to measure the strength of the association.

**Result:**

A total of 561 records (187 cases and 374 controls) were included from tuberculosis registers. In this study, the factors independently associated with unsuccessful tuberculosis treatment outcome were older age (AOR = 1.68, 95% CI: 1.142, 2.472), rural residence (AOR = 1.548, 95% CI: 1.055, 2.272), retreatment category (AOR = 2.12, 95% CI: 1.339, 3.357), underweight (BMI < 18.5 kg/m^2^) (AOR = 1.952, 95% CI: 1.240, 3.071), being HIV positive (AOR = 2.144, 95% CI: 1.372, 3.349) and having no treatment adherence support (AOR = 2.016, 95% CI: 1.270, 3.201).

**Conclusion and recommendation:**

In this study, socio-demographic, clinical, and treatment-related factors contributed to the risk of unsuccessful treatment outcomes. Targeted interventions should be taken into consideration to diminish poor tuberculosis treatment outcomes among high-risk groups throughout the whole tuberculosis treatment course.

## Background

Tuberculosis (TB) is an infectious disease that affects the parenchyma of the lungs (1, 2). It is still a serious public health issue around the world, and it is among the most deadly diseases (3). It is the major cause of morbidity, surpassing HIV/AIDS, and is one of the top ten causes of death worldwide (4). Unsuccessful or poor treatment outcome is defined as the number of TB patients who have treatment failure, defaulter, or death (5, 6). Monitoring tuberculosis treatment outcomes is crucial for determining the quality and improvement of TB treatments as well as identifying potential TB control challenges (7).

According to the World Health Organization (WHO) 2020 report, the global unsuccessful TB treatment outcome for patients receiving anti-TB treatment among new cases of TB is 15, and 24% for TB patients co-infected with HIV (4). On the other hand, a systematic review and meta-analysis in Africa indicated the overall pooled estimate of unsuccessful TB treatment outcome in Africa was reported to be 21.1% which is above the WHO-defined standard threshold of 15% with significant variation across countries (6). In Ethiopia, between 2017 and 2020 unsuccessful TB treatment rates ranged from 7.5 to 26% at the national level (8). According to recent studies in different parts of Ethiopia, the unsuccessful treatment outcome rate varies from place to place, with South East Ethiopia at 8.8% (9), West Ethiopia at 17.5% (10), and Southern Ethiopia at 14.5% (11).

Evidence from the scientific community revealed that unsuccessful TB treatment outcomes are an important reason for the development of medicine resistance tuberculosis (MDR-TB) (12). MDR-TB poses a significant therapy challenge as treating and curing patients is more difficult (13). Despite the fact that MDR-TB therapies have saved millions of lives around the world, a significant number of patients continue to die. Medicine-resistant tuberculosis killed over 214,000 people in 2018 (14). Most deaths and poor treatment outcomes are known to occur in the first months of MDR-TB treatment (15, 16). The other implication of unsuccessful TB treatment outcome is to lengthen the course of treatment term, which is due to the failure in treatment, so people continue to get treatment, and poor management can result in death (17).

Unsuccessful treatment outcomes may lead to a prolonged period of infection, drug resistance, and increased morbidity and mortality (18). Failure to complete treatments indicates a poor treatment outcome, which places a significant financial burden on the national budget. Tuberculosis can affect anyone, especially those who are still working or in their productive years. Approximately 75% of patients (15–50 years old) are in the most economically productive age range. On average, adult patients are expected to miss 3 to 4 months of work. If the patient dies, the firm will lose 15 years of earnings. These negative socioeconomic implications will be stronger in TB patients with poor treatment outcomes (19).

As long known by the WHO and the Federal Ministry of Health (FMOH), effective TB control is a challenge for the public health sector in high TB burden nations due to its constrained funding and coverage (20, 21). Despite the extension of the directly observed treatment short-course (DOTS) model and the public–private mix (PPM) have considerably enhanced Ethiopia’s TB control efforts over the past decade, TB remains a serious public health issue. Ethiopia achieved the millennium development goals for TB in 2015 and adopted a new post-2015 global End TB Strategy. The TB control program is currently being challenged by the spread of MDR-TB, which is the result of unsuccessful treatment outcomes (17, 20, 21). Therefore, the study aimed to assess the determinants of unsuccessful treatment outcomes among tuberculosis patients in Southwest Ethiopia regional state public hospitals.

## Methods and materials

### Study area study period

The study was conducted in Southwest Ethiopia regional state public hospitals. Bonga is the capital city of the region. The region has six zones with a population of 2,300,000. A total of ten (10) public hospitals are present in the region. The study was conducted in three selected public hospitals, namely, Bonga Gebretsadik Shawo Memorial General Hospital, Mizan Tepi University Teaching Hospital, and Tepi General Hospital. In the past 5 years, a total of 447, 520, and 407 TB patients were registered, respectively, in each hospital for tuberculosis treatment. The study was conducted from 1 August 2022 to 21 August 2022.

### Study design

A facility-based retrospective unmatched case–control study design was employed.

### Source population and study population

The source population were all registers of adult TB patients, who attended their course of treatment in Southwest Ethiopia regional state public hospitals from January, 2017, to December, 2021. Registers of TB patients with treatment outcomes of cured and treatment completed were study populations for controls.

### Case definition

**Cases:** TB patients with a TB treatment outcome reported or registered as treatment failure, default, and death during the course of anti-TB treatment (22).
**Controls:** TB patients with TB treatment outcomes reported as cured and treatment completed during the course of anti-TB treatment (5, 6).

#### Inclusion criteria

Records of all adult patients (18 and above) registered for TB treatment at the selected public hospitals from January 2017 to December 2021 and had known treatment outcomes.

#### Exclusion criteria

Patients with unknown treatment outcomes, incomplete medical records, and patients transferred to other health facilities.

#### Sample size determination

A two-population proportion formula using Epi-info version-7 statistical software for an unmatched case–control study design was used to estimate the sample size. According to the findings of a similar study conducted in eastern Ethiopia, lack of contact person, smear-negative pulmonary TB, smear positive at 2nd month of treatment initiation, and HIV-positive status were factors significantly associated with unsuccessful treatment outcome, and those variables were used to estimate the sample size (18). It is determined by using the following assumptions: the power of the study =80, 95% confidence level, the proportion of exposure among cases (p1), the proportion of exposure among controls (p2), and the ratio of cases to controls (r) =1:2 (Table 1). Of the four significant factors of an unsuccessful outcome, HIV-positive status gives the largest sample size. Therefore, the total sample size using the Fleiss w/cc method is 570 (190 cases and 380 controls).

**Table 1:** Socio-demographic characteristics of TB patients among cases and controls in Southwest Ethiopia regional state public hospitals, 2022 (*n* = 561).

Variables	Controls	Cases	Total (%)
	*n* = 374 (%)	*n* = 187 (%)	
Sex			
Male	225 (60.2)	101 (54)	326 (58.1)
Female	149 (39.8)	86 (46)	235 (41.9)
Age			
18–25	92 (24.6)	44 (23.5)	136 (24.2)
26–35	120 (32.1)	52 (27.8)	172 (30.7)
36–50	142 (38.0)	83 (44.4)	225 (40.1)
>50	20 (5.3)	8 (4.3)	28 (5.0)
Place of residence			
Urban	166 (44.4)	65 (34.8)	231 (41.2)
Rural	208 (55.6)	122 (65.2)	330 (58.8)
Contact person			
Yes	341 (91.2)	164 (87.7)	505 (90.0)
No	33 (8.8)	23 (12.3)	56 (10.0)
Source of contact person			
Family member	242 (71.0)	122 (74.4)	364 (72.1)
Neighbor	13 (3.8)	7 (4.3)	20 (4.0)
Health extension worker	72 (21.1)	29 (17.7)	101 (20.0)
Other healthcare worker	12 (3.5)	6 (3.7)	18 (3.6)
Friends	2 (0.6)	0 (0.0)	2 (0.4)
Family size (lived together)			
≤2	168 (44.9)	94 (50.3)	262 (46.7)
3–4	160 (42.8)	60 (32.1)	220 (39.2)
≥5	46 (12.3)	33 (17.6)	79 (14.1)

The formula is as follows (23):


$$\frac{r+1}{r}\frac{\dot{p}\left(1-\dot{p}\right){\left(2_{\beta }+z_{\alpha /2}\right)}^{2}}{{\left(p_{1}-p_{2}\right)}^{2}}$$

where *n* = sample size.

*r* = ratio of cases to controls.

$p^{*}$ = average proportion of exposed= $\frac{\left(p_{1}+p_{2}\right)}{2}$ .

$Z_{\beta }$ = the desired power of the study.

$Z_{\alpha /2}$ = the desired level of significance.

p1 = proportion of exposure among cases.

p2 = proportion of exposure among controls.

#### Sampling technique

A simple random sampling technique was employed to select three hospitals from the ten public hospitals found in the southwest Ethiopia regional state, namely, Bonga Gebretsadik Shao Memorial General Hospital (BGSMGH), Mizan Tepi University Teaching Hospital (MTUTH), and Tepi General Hospital (TGH) were selected. The number of study subjects (cases and controls) for each hospital is allocated based on total number of TB patients. According to the Health Management Information System (HMIS) report of each hospital, the number of TB patients who attend their course of TB treatment in the last 5 years (2017–2021) was 447 (64 cases and 383 control) at BGSH, 520 (72 cases and 448 controls) at MTUTH, and 407 (68 cases and 339 controls) at TGH.

The total sample size was proportionally allocated to each hospital based on the number of patients who attended TB treatment in the last 5 years: by using a proportional allocation formula $n_{i}=\frac{n}{N}\times N_{i}$ . Where, *n_i_* = the sample size for each hospital, *n* = total sample size to be selected, *N* = total number of TB patients in the selected three hospitals, and *N_i_* = a total number of TB patients in each hospital.

Therefore, 184 (60 cases and 124 controls) from BGSH, 213 (67 cases and 146 controls) from MTUTH, and 173 (63 cases and 110 controls) from Tepi General Hospital were selected.

### Data collection tools and procedure

The data were collected using a pretested data extraction checklist adapted from the tuberculosis (TB) registration logbook, which was developed by the Ministry of Health based on the World Health Organization (WHO) criteria. The checklist consists of socio-demographic factors (nine items), clinical-related characteristics (eleven items), and treatment or drug use-related factors (eight items). The data were collected from TB registries by three trained BSc Nurses working in the TB treatment unit. The data collection process was supervised by three trained onsite supervisors (one MSc nurse and two health officers). Every checklist was checked for its completeness during the data collection.

### Operational definition and terms

The World Health Organization TB definition and reporting framework (22) and the Ethiopian National Tuberculosis and Leprosy Control Program guidelines of TB Patients (24), definitions of Treatment outcomes were used in this study.

**Cured:** A bacteriologically confirmed TB patient who was subsequently smear- or culture-negative during the last month of treatment or on at least one previous occasion.
**Treatment Completed:** TB patient who just had treatment but did not meet the standards for cure or treatment failure.
**Treatment failure:** Patient receiving TB treatment whose sputum smear or culture proved positive at 5 months or later.
**Death:** TB patient who died in a hospital or health facility, or whose death is reported by the patient’s contact person (a family member, health extension worker, neighbor, or other healthcare workers) except death by accident.
**Loss to follow-up:** Tuberculosis patient whose treatment was interrupted for 2 consecutive months or more.
**New case:** Patients who had never been treated for TB or had taken anti-TB drugs for less than 1 month.
**Retreatment case:** individuals who had undergone prior TB therapy in any way (relapse, treatment after failure, or treatment after loss to follow-up).
**Body Mass Index (BMI)** is the patient’s weight in kilograms divided by the square of their height in meters. BMI < 18.5 kg/m^2^: underweight, BMI 18.5–24.9 kg/m^2^: normal, BMI 25–29.9 kg/m^2^: overweight, BMI 30 kg/m^2^ and above: obese (25).
**TB treatment adherence supporter:** A person who assists the patient in following the treatment schedule throughout treatment. It can be a health professional, health extension worker, or relative (26).

### Data quality assurance

To guarantee the quality of the data, a 1-day training session for data collectors and supervisors, a pre-test of the data extraction checklist, and ongoing supervision throughout the data collection were used. The main emphasis of the training was on the checklists, learning how to retrieve information from TB registries, and effective time management. Daily supervision was held in study settings by the onsite supervisor and principal investigator one time a week. The data extraction checklist was pretested on 5% of the sample (10 cases and 19 controls) at Wacha Hospital 1 week before the actual data collection. During the actual data collection, the supervisor reviewed the collected data each day to ensure its completeness and consistency. The data were re-checked during data entry, and any incomplete questionnaires were eliminated. The principal investigator then coded and entered the completed checklist into Epi Data version 4.6.

### Data processing and analysis

The data were checked for completeness, coded, and entered into Epi Data version 4.6 and exported to SPSS (Statistical Package for Social Science) version 26 for analysis. The status of unsuccessful treatment outcome (yes or no) was coded into 1 = cases and 0 = controls. Frequencies, means, standard deviation, and percentages were used for the descriptive analysis of data. Bivariate and multivariate logistic regression models were employed to identify the presence of association between the dependent and independent variables. Variables with a *p*-value *<*0.25 at 95% CI in the bivariate logistic regression were entered into a multivariate logistic regression model after checking whether the necessary assumptions were fulfilled.

Finally, predictor variables with a *p*-value of less than 0.05 in the multivariate logistic regression model were taken as statistically significant risk factors for unsuccessful tuberculosis treatment outcomes. The odds ratio and 95% confidence level were used to measure the strength of the association. Model fitness was checked by using Hosmer and Lemeshow’s goodness-of-fit test. Multivariate analysis was considered for control of confounders. Finally, the findings were presented in the form of tables and text.

### Ethical consideration

An ethical clearance letter was obtained from the institutional review board (IRB) of Jimma University. Supportive letters were written to the selected hospitals for permission and cooperation before data collection. Approval to retrieve data from hospitals’ data record systems were obtained from the clinical director and the concerned bodies of each hospital. As this is a retrospective study, the consent of patients was not obtained. However, patient information was handled anonymously. Thus, to assure confidentiality the name of the patients was not recorded in the data extraction checklist rather the identifiers of patients who participated in the study was coded and data were kept secure.

## Result

### Socio-demographic characteristics

A total of 561 records, consisting of 187 (33.3%) cases and 374 (66.6%) controls, were analyzed in this study. As most of the patients’ basic information had been missed, nine (3 cases and 6 controls) registers were removed from the data entry process. In the entire sample used for analysis, 326 (58.1%) of the participants were male, and the majority of the patients 225 (40.1%) were in the age group of 36–50 years, with a mean age of 33.39 (±9.9). Regarding the place of residence, 65.2% of patients were rural residents among cases with a comparable variation of 55.6% in controls (Table 2).

**Table 2:** Clinical characteristics of TB patients among cases and controls in Southwest Ethiopia regional state public hospitals, 2022 (*n* = 561).

Patient characteristics	Category	Controls	Cases	Total *N* (%)
Source of initial referrals	Health extension worker	64 (17.1)	24 (12.8)	88 (15.7)
	Other health facility	9 (2.4)	6 (3.2)	15 (2.7)
	Self	301 (80.5)	157 (84)	458 (81.6)
TB treatment outcome	Treatment failure	0 (0.0)	51 (27.3)	51 (9.1)
	Lost to follow-up	0 (0.0)	76 (40.6)	76 (13.5)
	Death	0 (0.0)	60 (32.1)	60 (10.7)
	Cured	185 (49.5)	0 (0.0)	185 (33.0)
	Treatment completed	189 (50.5)	0 (0.0)	189 (33.7)
Initial patients’ category	New	294 (78.6)	128 (68.4)	422 (75.2)
	Relapse	6 (1.6)	19 (10.2)	25 (4.5)
	Return after failure	7 (1.9)	12 (6.4)	19 (3.4)
	Return after default	42 (11.2)	18 (9.6)	60 (10.7)
	Transfer in	25 (6.7)	10 (5.3)	35 (6.2)
Form of TB	Smear positive (PTB+)	182 (48.7)	70 (37.4)	252 (44.9)
	Smear negative (PTB−)	167 (44.7)	103 (55.1)	270 (48.1)
	Extra pulmonary	25 (6.7)	14 (7.5)	39 (7.0)
Second-month AFB	Positive	31 (16.6)	44 (60.3)	75 (28.8)
	Negative	156 (83.4)	25 (34.2)	181 (69.6)
	Not checked	0 (0.0)	4 (5.5)	4 (1.5)
Fifth-month AFB	Positive	0 (0.0)	45 (95.7)	45 (19.2)
	Negative	187 (100.0)	0 (0.0)	187 (79.9)
	Not checked	0 (0.0)	2 (4.3)	2 (0.9)
Sixth−/eighth-month AFB	Positive	0 (0.0)	30 (93.8)	30 (13.7)
	Negative	151 (80.7)	0 (0.0)	151 (68.9)
	Not checked	36 (19.3)	2 (6.3)	38 (17.4)
HIV AIDS status	Positive	66 (17.6)	54 (28.9)	120 (21.4)
	Unknown	22 (5.9)	12 (6.4)	34 (6.1)
	Negative	286 (76.5)	121 (64.7)	407 (72.5)
Baseline weight in kg	≤40	2 (0.5)	1 (0.5)	3 (0.5)
	≥55	245 (65.5)	114 (61.0)	359 (64.0)
	41–54	127 (34.0)	72 (38.5)	199 (35.5)
Body mass index	<18.5 kg/m^2^	62 (16.6)	46 (24.6)	108 (19.3)
	≥25 kg/m^2^	5 (1.3)	6 (3.2)	11 (2)
	18.5–24.9 kg/m^2^	307 (82.1)	135 (72.2)	442 (78.8)
Co-morbidity	Diabetes	25 (6.7)	9 (4.8)	34 (6.1)
	Hypertension	9 (2.4)	8 (4.3)	17 (3.0)
	Other	10 (2.7)	7 (3.7)	17 (3.0)
	None	330 (88.2)	163 (87.2)	493 (87.9)

Other, asthma, CVD, and malnutrition; AFB, acid-fast bacilli; TB, tuberculosis; PTB+, smear-positive pulmonary TB; PTB−, smear-negative positive pulmonary TB.

### Clinical characteristics of patients

Out of the 561 samples, 458 (81.6%) were self-referrals. Regarding the TB treatment outcome, among cases (*n* = 187) 40.6% were recorded as “default,” whereas 189 (50.5%) of controls (*n* = 374) were treatment completed. A higher proportion of the retreatment category was in the case group than the control group (26.2% vs. 15.0%). The majority of study subjects in the case group, i.e., 103 (55.1%), were smear-negative PTB. Among the patients included, a significantly higher proportion of study subjects in the case group than the control group were HIV positive, i.e., 28.9% vs. 17.6%. Moreover, 442 (78.8%) of all included patients had a normal range of body mass index (18.5–24.9 kg/m^2^) (Table 3).

**Table 3:** Treatment-related characteristics of TB patients among cases and controls in Southwest Ethiopia regional state public hospitals, 2022 (*n* = 561).

Patient characteristics	Category	Controls	Cases	Total *N* (%)
Year of treatment	Before 2019	154 (41.2)	73 (39.0)	227 (40.5)
	2019 and after	220 (58.8)	114 (61.0)	334 (59.5)
Center of treatment	MTUTH	147 (39.3)	66 (35.3)	213 (38.0)
	BGSMH	117 (31.3)	59 (31.6)	176 (31.4)
	TGH	110 (29.4)	62 (33.2)	172 (30.7)
Type of anti-TB	2RHZE/4RH	323 (86.4)	159 (85.0)	482 (85.9)
	2SRHZE/1RHZE/5RHE	7 (1.9)	10 (5.3)	17 (3.0)
	2RHZ/4RH	18 (4.8)	5 (2.7)	23 (4.1)
	Others	26 (7.0)	13 (7.0)	39 (7.0)
HAART started (HIV+)	Yes	66 (100.0)	52 (96.3)	118 (98.3)
	No	0 (0.0)	2 (3.7)	2 (1.7)
CPT started for HIV+	Yes	52 (78.8)	46 (85.2)	98 (81.7)
	No	14 (21.2)	8 (14.8)	22 (18.3)
Adherence support	Yes	315 (84.2)	141 (75.4)	456 (81.3)
	No	59 (15.8)	46 (24.6)	105 (18.7)
Support provided at	Hospital (HPs)	229 (72.7)	96 (68.1)	325 (71.3)
	Health post (HEW)	86 (27.3)	45 (31.9)	131 (28.7)
Drug adverse reaction(s)	Itchy skin/skin rash	11 (2.9)	6 (3.2)	17 (3.0)
	GI upset (N/V, D)	17 (4.5)	9 (4.8)	26 (4.6)
	Numbness of hands/legs	1 (0.3)	0 (0.0)	1 (0.2)
	Hepatotoxicity	2 (0.5)	2 (1.1)	4 (0.7)
	Other	4 (1.1)	1 (0.5)	5 (0.9)
	None	339 (90.6)	169 (90.4)	508 (90.6)

MTUTH, Mizan Tepi University Teaching Hospital; BGSMH, Bonga Gebretsadik Shawo Memorial Hospital; TGH, Tepi General Hospital; HAART, highly active anti-retroviral therapy.

### Treatment-related characteristics of patients

Among the patients included, the majority 482 (85.9%) were treated with anti-TB treatment of 2RHZE/4RH. A higher proportion of patients had no treatment adherence support in case groups when compared to control groups, i.e., 46 (24.6%) vs. 59 (15.8%) (Table 4).

**Table 4:** Bivariate and multivariate analysis of factors associated with unsuccessful tuberculosis treatment outcome among cases and controls in Southwest Ethiopia regional state public hospitals, 2022 (*n* = 561).

Patient characteristics	Controls	Cases	COR (95% CI)	*p*-value	AOR (95% CI)	*p*-value
	*n* = 374	*n* = 187				
Sex						
Male	225 (60.2)	101 (54.)	Ref			
Female	149 (39.8)	86 (46)	1.286 (0.902, 1.833)	0.164	1.408 (0.962, 2.059)	0.078
Age						
≤35	234 (62.6)	99 (52.9)	Ref			
>35	140 (37.4)	88 (47.1)	1.486 (1.041, 2.120)	0.029	1.680 (1.142, 2.472)	0.008
Place of residence						
Urban	166 (44.4)	65 (34.8)	Ref			
Rural	208 (55.6)	122 (65.2)	1.498 (1.041, 2.155)	0.029	1.548 (1.055, 2.272)	0.026
Contact person						
Yes	341 (91.2)	164 (87.7)	Ref			
No	33 (8.8)	23 (12.3)	1.449 (0.824, 2.547)	0.197	1.551 (0.835, 2.883)	0.165
Family size						
≤2	168 (44.9)	94 (50.3)	Ref			
3–4	160 (42.8)	60 (32.1)	0.670 (0.454, 0.989)	0.044	0.733 (0.481, 1.117)	0.149
≥5	46 (12.3)	33 (17.6)	1.282 (0.767, 2.143)	0.343	1.128 (0.633, 2.009)	0.683
Source referrals						
HEW	64 (17.1)	24 (12.8)	Ref			
HF	9 (2.4)	6 (3.2)	1.778 (0. 0.572, 5.528)	0.320	1.629 (0.434, 6.112)	0.470
Self	301 (80.5)	157 (84.0)	1.391 (0.838, 2.310)	0.202	1.480 (0.844, 2.594)	0.171
Patients’ category						
New	293 (78.3)	128 (68.4)	Ref			
Retreatment	56 (15.0)	49 (26.2)	2.003 (1.295, 3.098)	0.002	2.120 (1.339, 3.357)	0.001
Transfer in	25 (6.7)	10 (5.3)	0.916 (0.427, 1.962)	0.821	1.634 (0.695, 3.839)	0.260
Form of TB						
Smear positive	182 (48.7)	70 (37.4)	Ref			
Smear negative	167 (44.7)	103 (55.1)	1.604 (1.109, 2.320)	0.012	1.475 (0.995, 2.187)	0.053
Extra pulmonary	25 (6.7)	14 (7.5)	1.456 (0.716, 2.961)	0.300	1.411 (0.652, 3.052)	0.382
Co-morbidity						
Diabetes	25 (6.7)	9 (4.8)	0.729 (0.333, 1.597)	0.429	0.541 (0.234, 1.253)	0.152
Hypertension	9 (2.4)	8 (4.3)	1.80 (0.682, 4.750)	0.235	1.632 (0.576, 4.624)	0.357
Other	10 (2.7)	7 (3.7)	1.417 (0.530, 3.791)	0.487	1.984 (0.695, 5.666)	0.201
None	330 (88.2)	163 (87.2)	Ref			
HIV AIDS status						
Positive	66 (17.6)	54 (28.9)	1.934 (1.274, 2.937)	0.002	2.144 (1.372, 3.349)	0.001
Unknown	22 (5.9)	12 (6.4)	1.289 (0.618, 2.688)	0.498	1.312 (0.605, 2.844)	0.492
Negative	286 (76.5)	121 (64.7)	Ref			
Body mass index						
<18.5 kg/m^2^	62 (16.6)	46 (24.6)	1.834 (1.20, 2.802)	0.005	1.952 (1.240, 3.071)	0.004
≥25 kg/m^2^	5 (1.3)	6 (3.2)	0.91 (0.174, 4.747)	0.911	1.042 (0.186, 5.830)	0.963
18.5–24.9 kg/m^2^	307 (82.1)	135 (72.2)	Ref			
Adherence support						
Yes	315 (84.2)	141 (75.4)	Ref			
No	59 (15.8)	46 (24.6)	1.742 (1.129, 2.687)	0.012	2.016 (1.270, 3.201)	0.003
Type of anti-TB						
2RHZE/4RH	323 (86.4)	159 (85.0)	Ref			
2SRHZE/1RHZE//5RHE	7 (1.9)	10 (5.3)	2.902 (1.084, 7.766)	0.034	2.219 (0.730, 6.744)	0.160
2RHZ/4RH	18 (4.8)	5 (2.7)	0.564 (0.206, 1.548)	0.266	0.460 (0.142, 1.495)	0.197
Others	26 (7.0)	13 (7.0)	1.016 (0.508, 2.030)	0.965	0.000 (0.000)	1.00

COR, crude odds ratio; AOR, adjusted odds ratio; E—ethambutol; H—isoniazid; R—rifampicin; S—streptomycin; Z—pyrazinamide. Hosmer and Lemeshow’s test, 0.509; Ref, reference group.

## Discussion

Designing a suitable evidence-based system to mitigate morbidity and mortality can be made easier by understanding the factors related to unsuccessful treatment outcomes. Age over 35 years, residing in rural areas, the retreatment category, being underweight (BMI <18.5 kg/m^2^), having HIV infection, and having no support for treatment adherence were all identified by this study to be predictors of unsuccessful TB treatment outcomes.

In this study, it was found that patients of older age (> 35 years) were more likely to encounter unsuccessful treatment outcomes than younger age. This is consistent with earlier research studies that were carried out in different Ethiopian parts: Arba Minch (27), Gambela (28), Debretabour (29), Gibe Woreda, Southern Ethiopia (11), and West Ethiopia (10). Various findings from around the world also support this finding, in Mozambique (30), Malawi (31), Anqing, China (32), Kepong district, Kuala Lumpur, Malaysia (33), Bahawalpur, Pakistan (34) and South Korea (35). More treatments are failing because, as people age, their chances of developing concurrent diseases (such as malnutrition, diabetes, and cancer) increase. Additionally, as people age, their bodies generally deteriorate biologically and physiologically, which can nullify their immune systems and increase rates of death in the aging population (36–38). Another possible explanation might be because older patients are more prone to experience treatment interruptions as they are relatively frailer, non-ambulatory, and dependent on family members to obtain to medical facilities for their continuous care.

According to this study, being a rural resident increases the risk of unsuccessful treatment outcomes as compared to patients’ residence in an urban area. This was similar to other findings in Arba Minch (27), East Wollega (39), and Gambela (28). This may be due to the fact that patients from rural areas may not be as familiar well with the disease and its treatments. In addition, longer travel distances to treatment facilities and related expenditures may force patients to interrupt or stop their treatments (40–43). In contrast, a study in Debretabour found that patients living in urban areas had more unsuccessful treatment outcomes than rural residents. This was explained by the relatively higher co-morbidities (including HIV and diabetes mellitus) and other addiction-related factors among urban residents (29). The dissimilarity in findings might be attributed in part to differences in study settings, which could account for variations in socioeconomic and healthcare infrastructure. Another possible reason for this variation might be the time of study period and the statistical pooling effect of the reference groups.

In the current study, patients with a history of previous tuberculosis treatment had a higher risk of developing an unsuccessful TB treatment outcome. This is supported by research done in Arba Minch (27), Gambela (28), East Wollega (39), Ghana (26), South Africa (44), Kilifi County Kenya (45), Malaysia (46), Bahawalpur, Pakistan (34), Portugal (47), Thailand (48), and İstanbul Turkey (37). These observations could be explained by the fact that because the patients were continuously exposed to anti-TB medication, suboptimal therapy and drug resistance may have occurred. As a result, previous failures may have been caused by drug resistance, and patients who have previously defaulted are likely to have poor compliance or drug resistance (37, 49). The second possible explanation might be that patients who have a history of previous therapy need more extensive care, which could be the reason for discontinuing treatment (40, 50).

Additionally, patient behavior may have played a role in the unsuccessful outcome; a study found that patients who had previously been lost to follow-up may be reluctant and more likely to discontinue their treatment (51). Overall, patients who had previously failed treatment were generally more likely to fail a treatment, and patients who defaulted on previous therapy had a higher rate of default in retreatment (52). Such patients should receive the necessary attention to ensure that they complete the entire course of treatment, and if they have already experienced a treatment failure, an early referral should be made for MDR screening.

Being HIV positive was also significantly associated with unsuccessful TB treatment outcomes. According to the result of this study, patients who were HIV positive were two times higher risk of experiencing unsuccessful treatment outcomes. This is supported by other studies done in Arba Minch (27), Blue Hora General Hospital (53), Eritrea (54), Malawi (31), Mozambique (55), Kepong district, Kuala Lumpur, Malaysia (46), and Portugal (47). This can be explained by HIV and TB co-infection can weaken the immune system, which has a detrimental impact on treatment outcomes and can even result in death. It is clear that HIV AIDS continues to constitute a dual problem, raising both the risk of contracting TB and the poor outcome of the treatment. It might also be because taking both antiretroviral and anti-TB medications daily can be difficult and challenging for a patient, which could lead to therapeutic failure. Mortality from co-infections is higher than mortality from TB infection alone.

The body mass index (BMI) of patients was found to be significantly associated with poor outcomes. According to this study, the odds of underweight (BMI <18.5 kg/m^2^) were more likely to develop unsuccessful TB treatment outcomes when compared to patients who had normal body mass index (18.5–24.9 kg/m^2^). This is in line with other studies done in Addis Ababa (56), Mozambique (57), and Taiwan, Taipei (58). It is possible that the lower immunity and more severe TB infection in this population are to blame for the higher death rate among underweight TB patients. Underweight patients may experience a higher burden of tuberculosis infection and more severe TB disease due to suppressed lymphocyte activation and reduced Th1 cytokine secretions (59).

Generally, there is a two-way interaction between TB and malnutrition. Malnutrition raises the risk of contracting severe TB diseases and has a negative impact on treatment outcomes. Severe TB disease also causes patients to be underweight. Therefore, for a better treatment outcome in undernourished TB patients, enhanced nutritional surveillance and the early provision of nutrition support are crucial (60). Contradicting this finding, a study in Kenya showed that in the first 3 months of tuberculosis treatment, being overweight was associated with unsuccessful treatment outcomes (45). This dissimilarity could be explained by differences in socioeconomic background of the study settings.

In addition, patients’ treatment adherence support was significantly associated with unsuccessful treatment outcomes. According to the current study, patients with no treatment adherence support were 2.01 times more likely to develop unsuccessful treatment outcomes. This can be possibly explained by having no treatment adherences support, who assists the patients in maintaining the treatment schedule throughout the course of treatment can result for drug interruption and lost to follow-up and/or treatment failure.

### Limitation of the study

The potential limitation of this study was the use of secondary data, which is solely restricted to what was recorded in the hospital TB records. Due to this, several variables, such as socioeconomic characteristics (employment, profession, and income), behavioral factors, and other important factors, were not easily accessible.

## Conclusion

This study provides useful insights into factors associated with unsuccessful treatment outcomes. Based on the current study older age, living in a rural area, retreatment category, being underweight, being HIV positive, and having no support for treatment adherence were found to be contributing factors for unsuccessful treatment outcomes. Therefore, emphasis and strict attention are required for patients with those high-risk groups to mitigate poor outcomes throughout the whole TB treatment course. Therefore, hospitals (health professionals) should make frequent supportive care and health education programs about the disease and its treatment, providing nutritional support (therapeutic food) and a combination of medical interventions and social support should be taken into account for HIV-positive and patients of rural residents. In addition, further researchers should conduct prospective and longitudinal studies to identify the causal relationship between unsuccessful treatment outcomes and associated factors.

## Data Availability

The data sets presented in this article are not readily available because of privacy and ethical concerns. However, reasonable requests to access the datasets should be directed to solomonber32@gmail.com.
